# Treatment With Valoctocogene Roxaparvovec in a Patient With Severe Hemophilia A Led to Sustained Normal FVIII Levels

**DOI:** 10.1111/hae.70261

**Published:** 2026-03-12

**Authors:** Kerstin Herbst, Behnaz Pezeshkpoor, Claudia Klein, Thilo Albert, Philipp Lutz, Christian Strassburg, Ulrich Spengler, Georg Goldmann, Johannes Oldenburg

**Affiliations:** ^1^ Institute of Experimental Hematology and Transfusion Medicine University of Bonn Bonn Germany; ^2^ Department of General Internal Medicine I University of Bonn Bonn Germany; ^3^ Center For Rare Diseases Bonn (ZSEB) University Clinic Bonn Bonn Germany

**Keywords:** alanine transaminase, gene therapy, hemophilia A, treatment outcome, valoctocogene roxaparvovec

## Abstract

**Introduction:**

Valoctocogene roxaparvovec, an adeno‐associated virus (AAV)‐based gene therapy, enables endogenous factor VIII (FVIII) expression in patients with severe hemophilia A without the need for regular FVIII infusions. Long‐term follow‐up assesses durability, safety, and immune‐related challenges following gene therapy.

**Aim:**

Evaluation of six years of post‐infusion outcomes of a patient receiving valoctocogene roxaparvovec, focusing on FVIII expression, immune response management, and adverse events (AEs).

**Methods:**

A 44‐year‐old male with severe hemophilia A received a single infusion of valoctocogene roxaparvovec (6 × 10^13^ vector genomes/kg) in the GENEr8‐1 study. Follow‐up assessments included FVIII levels, liver function tests, and immune suppression management.

**Results:**

Post‐infusion, the patient experienced an initial ALT elevation and FVIII decline, requiring oral prednisolone (60 mg/day) at week five. ALT elevation grade 3 (peaking at 555 U/L) was treated with intravenous methyl‐prednisolone (week 6), followed by oral prednisolone. To mitigate cortisone side effects, prednisolone was switched to budesonide therapy at week 19 and continued for 6 months. FVIII activity (measured with a chromogenic FVIII assay) reached a peak of 202 IU/dL at month 6 and declined subsequently to levels of 50–70 IU/dL. After six years, FVIII activity remained normal, thus eliminating prophylactic FVIII infusions and bleeding episodes. A total of 20 AEs occurred, including two serious adverse events related to ALT elevation and a traumatic acetabulum fracture.

**Conclusion:**

Valoctocogene roxaparvovec can offer durable FVIII expression in severe hemophilia A. This case provides valuable insights into personalised immunosuppression therapy with a favourable outcome for long‐term efficacy and safety.

## 1 Introduction

Hemophilia A is a hereditary bleeding disorder characterised by a deficiency of clotting factor VIII (FVIII), leading to recurrent hemorrhages [1]. Severe hemophilia A is characterised by FVIII activity levels below 1 IU/dL, causing spontaneous bleeding episodes. Traditional management strategies, especially prophylactic FVIII replacement therapies, have substantially improved patient outcomes. However, these treatments are associated with challenges such as regular intravenous administrations, the prevention of joint arthropathy, the development of inhibitory antibodies, and considerable healthcare costs [2]. In recent years, alternative therapies such as monoclonal antibodies mimicking FVIII or, most recently, monoclonal anti‐TFPI antibodies with a pro‐coagulant balance of blood coagulation have become available for the treatment of hemophilia in patients with and without inhibitors, leading to a reduced treatment burden [3, 4]. Recent advancements in gene therapy offer another promising alternative by inducing long‐term expression of clotting FVIII [5, 6].

Valoctocogene roxaparvovec (AAV5‐hFVIII‐SQ) represents a novel adeno‐associated virus (AAV)‐based gene therapy designed to deliver a functional copy of the F8 gene to patients with severe hemophilia A [7]. By enabling endogenous production of FVIII, valoctocogene roxaparvovec aims to provide efficient therapeutic levels of the clotting, thereby reducing or eliminating the need for regular infusions [8, 9]. The mechanism of action of valoctocogene roxaparvovec involves the use of a non‐replicating AAV vector to deliver the B‐domain‐deleted F8 transgene directly to hepatocytes [8]. Upon successful transduction, hepatocytes produce and secrete functional FVIII protein [10]. Clinical trials have demonstrated promising efficacy profiles, with significant increases in FVIII levels and reduced bleeding episodes [8, 9, 11]. Moreover, the therapy demonstrated a favourable safety profile, with most adverse events (AEs) being associated with mild to moderate Aspartate aminotransferase (AST) / Alanine aminotransferase (ALT) elevation is suggestive of being immune mediated [8, 9, 12]. These trials suggest that valoctocogene roxaparvovec could offer a long‐term treatment for hemophilia A patients, mitigating the burdens associated with conventional therapies.

Challenges regarding hepatotoxicity [13, 14, 15], immune‐mediated clearance of transduced cells [16], and the durability of transgene expression are addressed in follow‐up studies [8, 9, 17]. The variability in patient responses observed in hemophilia gene therapy trials indicates the need for personalised approaches to optimise therapeutic outcomes [17].

This report presents a six‐year follow‐up of a patient with severe hemophilia A who received valoctocogene roxaparvovec, highlighting both the long‐term efficacy with normal FVIII levels and the personalised immunosuppression management following the gene therapy infusion.

## 2 Material and Methods

### 2.1 Patient Description

A 44‐year‐old male with severe hemophilia A caused by an intron 22 inversion presented for treatment with valoctocogene roxaparvovec as part of the GENEr8‐1 study [9]. The patient provided informed consent in accordance with the Declaration of Helsinki for the study duration and the follow‐up study. His medical history included the intake of carbamazepine since 1992 for the prevention of epileptic seizures, as well as resolved hepatitis B and C infections. He had no clinically relevant arthropathy and maintained a healthy liver.

The patient was receiving a prophylactic treatment with an extended half‐life (EHL) FVIII product, administered at 12 IU/kg twice weekly, and it was continued for four weeks after gene therapy. The calculated half‐life for the FVIII product was within the expected range.

For the gene therapy, the patient received a single infusion of valoctocogene roxaparvovec at a dose of 6 × 10^13^ vector genomes per kilogram within the GENEr8‐1 study program [9]. The infusion was completed without any complications, and no adverse reactions were reported. Liver function and coagulation parameters were monitored according to the study protocol, including initial weekly testing of ALT, AST, and FVIII:C levels.

### 2.2 Coagulation Parameter Assessments

For assessing the FVIII activity (FVIII:C), citrated plasma was obtained by centrifugation of blood samples at 2500 × *g* for 15 min and either directly assayed or stored in aliquots at ≤−40°C until use. FVIII activities were determined on an Atellica Coag 360 system (Siemens Healthineers, Forchheim, Germany) using two different assays: an FVIII one‐stage assay (OSA) based on Actin FS (Siemens) and an FVIII chromogenic substrate assay (CSA) based on bovine FVIII (Siemens). All FVIII assays were calibrated down to 1 IU/dL using standard human plasma (Siemens) [18]. FVIII inhibitors were monitored as previously described with modifications [19, 20]. D‐dimer was determined on an automated coagulation analyser (Atellica Coag 360) using the INNOVANCE D‐dimer assay (both Siemens Healthineers). Liver enzymes, including gamma‐glutamyl transferase (GGT), ALT, and AST levels, were determined in plasma in units per litre (U/L) on a Cobas c702 (Roche Diagnostics) using the ASTPM reagents (Roche Diagnostics). Reference values provided by the laboratory were as follows: for GGT <60 U/L; for ALT <50 U/L; for AST <50 U/L.

## 3 Results

### 3.1 Post‐Infusion Course and Initial Immune Suppressive Therapy

Five weeks following the valoctocogene roxaparvovec infusion, the patient exhibited an increase in ALT levels by more than eight‐fold above baseline (baseline: 32 U/L), accompanied by a significant decline in FVIII:C levels (FVIII:C CSA/OSA 32/53 IU/dL at week 5 to 18/28 IU/dL at week 6, respectively). In response to these changes, an oral prednisolone regimen was initiated at 60 mg daily to mitigate immune‐mediated clearance of transduced hepatocytes and control potential vector‐induced hepatic inflammation.

**FIGURE 1:**
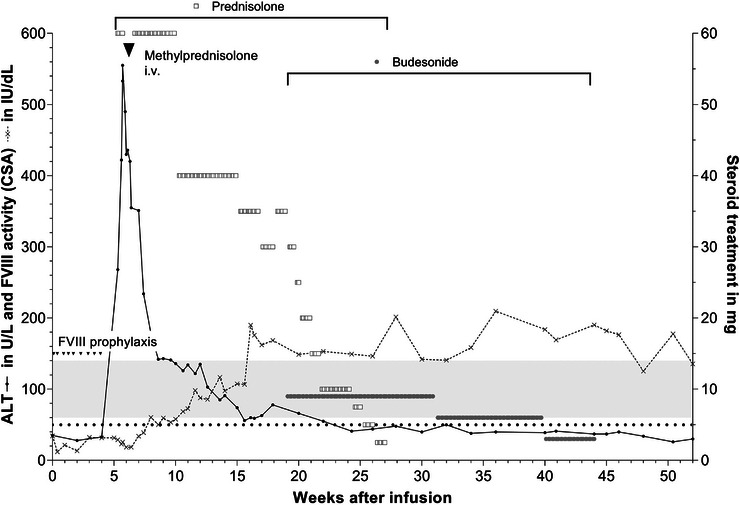
FVIII activity, ALT levels and immune suppression in the first year after valoctocogene roxaparvovec infusion. Initial immunosuppression consisted of prednisolone (grey open squares) at 60 mg daily. Following a grade 3 ALT elevation, the patient was hospitalised and treated with intravenous methyl‐prednisolone (black arrow) at 1 g/day for 3 days, followed by a tapering regimen of 500 mg daily for 2 days and 125 mg daily for another 2 days. Prednisolone was then reinitiated at 60 mg daily and continued until week 10. Tapering was carried out in 5 mg increments down to 10 mg, followed by 2.5 mg steps. Budesonide (grey‐filled dots) was introduced at week 19 at a dose of 3 mg three times daily. Tapering began at week 31, with the dose reduced to 3 mg twice daily and subsequently to 3 mg once daily. The course of ALT (U/L, black dots with solid line) and FVIII:C (IU/dL, CSA, crosses with dotted line) measurements over the first year is shown, with both plotted on the left *y*‐axis. Steroid treatment doses (prednisolone and budesonide) are plotted on the right *y*‐axis. The black triangles represent FVIII infusions. The black dotted line indicates the ALT reference value of 50 U/L, while the grey shaded area represents the reference range for CSA FVIII:C (60–140 IU/dL). ALT: alanine transaminase; CSA: chromogenic substrate assay; FVIII:C ‐ FVIII activity.

Despite prednisolone treatment, ALT levels continued to increase, reaching a peak of 555 U/L (17‐fold above baseline). The patient was hospitalised and received intravenous methyl‐prednisolone therapy for seven days (1 g/day for 3 days, 500 mg/day for 2 days and 125 mg/day for 2 days) to control the presumed immune response to the AAV capsid and prevent further elevation of liver enzymes. Following this course, ALT levels decreased, and oral prednisolone was reinstated with an initial dose of 60 mg (Figure 1). The patient was discharged after seven days.

### 3.2 Immune Suppression Regimen With Prednisolone and Budesonide

Following the high‐dose methyl‐prednisolone therapy, the patient was maintained on oral prednisolone (60 mg/day) for 24 days, with a gradual tapering schedule until week 27 post‐infusion. During the tapering phase at week 18, a rise of ALT levels (2.4‐fold above baseline) occurred at a prednisolone dose of 30 mg. In response to this relapse, the prednisolone dosage was increased to 35 mg/day for one week to regain control over hepatic enzyme elevations.

At week 18 budesonide (3 × 3 mg/day) was introduced due to significant side effects of the long‐term prednisolone therapy (thrush oral mucosa) and continued for 12 weeks. Following the start of budesonide therapy, prednisolone was tapered off over a period of eight weeks. The budesonide regimen was maintained at 9 mg/day until week 31 and then tapered to 6 mg/day for nine weeks, followed by 3 mg/day for four weeks, and finally discontinued by week 44. During this time, the liver enzyme levels returned to the normal range (Figure 1).

### 3.3 Long‐Term Follow‐Up and Durability of Normal FVIII Levels

**FIGURE 2:**
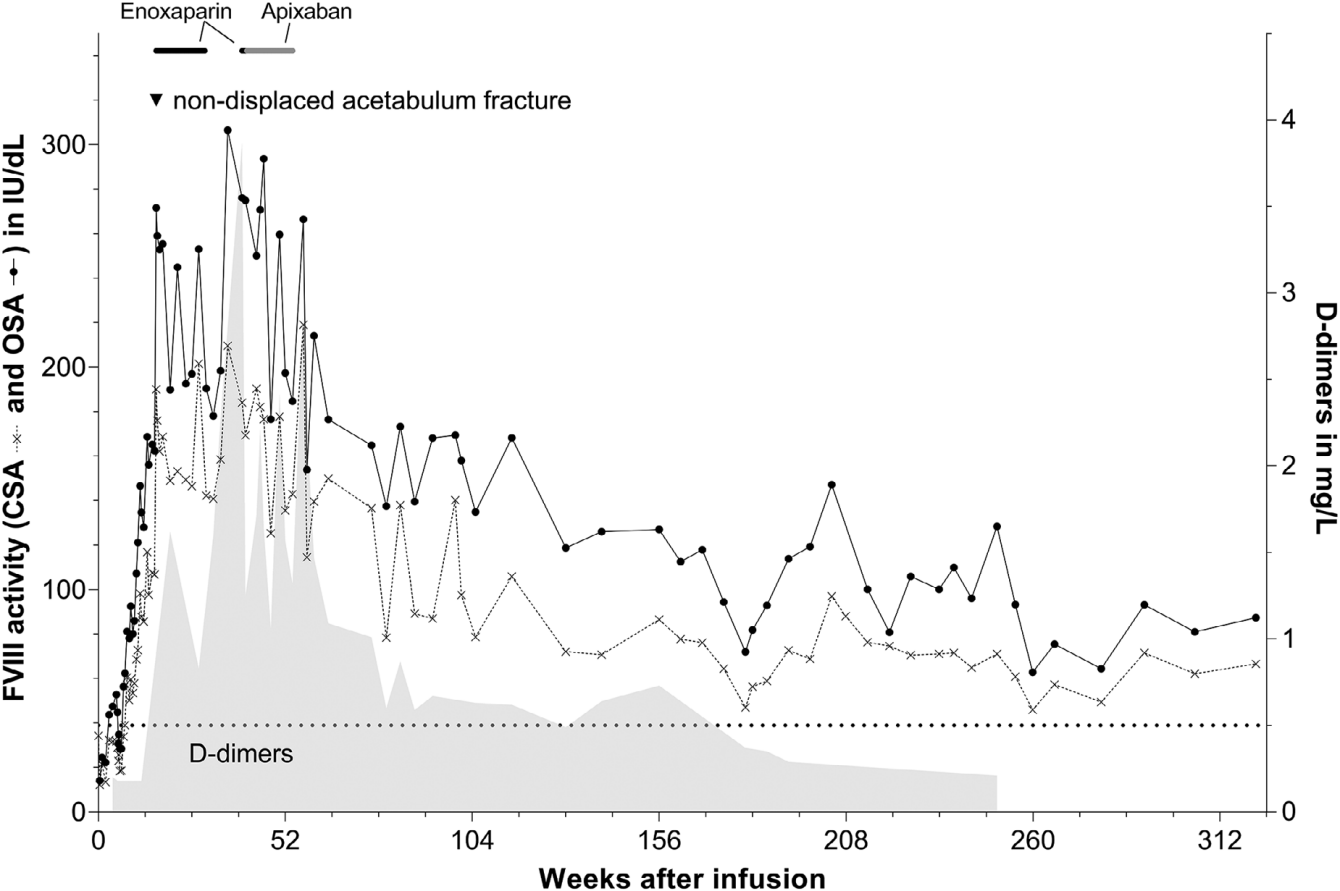
FVIII activity, D‐dimer levels, and anticoagulant treatment were monitored over a follow‐up period of more than six years. The course of FVIII:C levels (IU/dL, measured by CSA (crosses with dotted line) and OSA (black dots with solid line)) is plotted on the left *y*‐axis, while D‐dimer levels (mg/L, shown in grey shaded) are plotted on the right *y*‐axis. Anticoagulant therapy with enoxaparin (40 mg/day, shown in black dots) was initiated in week 16 due to a non‐displaced acetabulum fracture and administered until week 30. An elevation in D‐dimer levels at week 40 prompted the re‐initiation of anticoagulant therapy with enoxaparin (40 mg/day, black dots, weeks 40–41) followed by apixaban (2.5 mg twice daily, grey dots, weeks 41–54). The black dotted line represents the D‐dimer reference value of 0.5 mg/L. CSA: chromogenic substrate assay; FVIII:C ‐ FVIII activity; OSA: one‐stage assay.

**FIGURE 3:**
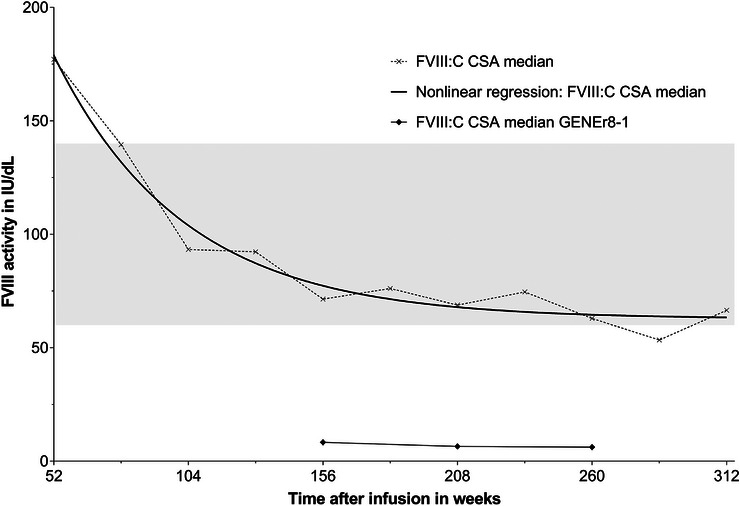
Median FVIII activity levels compared to the median from the GENEr8‐1 clinical study. The median FVIII:C levels (measured by CSA, grey crosses with dotted line) were calculated at 26‐week intervals. The solid black line represents the non‐linear regression of these median values, while the grey shaded area indicates the CSA reference range (60–140 IU/dL). The black rectangles correspond to data published by Leavitt et al., which represent median FVIII CSA levels from the GENEr8‐1 study cohort (*n* = 132) [21]. CSA: chromogenic substrate assay; FVIII:C ‐ FVIII activity.

Throughout the follow‐up period, the patient's FVIII levels were regularly monitored to assess the efficacy of the gene therapy based on CSA and OSA FVIII assays (Figure 2). Following a peak at month eight post‐infusion—reaching 210 IU/dL FVIII:C by CSA and 307 IU/dL by OSA (with OSA values consistently about 1.6 times higher than CSA throughout the observation period, Figure 2)—levels declined to 136 IU/dL CSA and 197 IU/dL OSA, respectively, by month 12. During the subsequent follow‐up years, FVIII levels fluctuated (year 2: CSA 87–219 IU/dL, year 3: CSA 71–106 IU/dL, year 4: CSA 47–97 IU/dL, year 5: CSA 46–76 IU/dL, year 6: CSA 49–72 IU/dL). The median values for 26‐week intervals were calculated, and non‐linear regression analysis was performed to assess the long‐term sustainability of FVIII expression. These data were compared to the five‐year follow‐up of the GENEr8‐1 clinical trial (Figure 3) [21]. Overall, the patient shows significantly higher FVIII levels compared to the median CSA data from the clinical study. The data also indicate that the FVIII levels for the patient have apparently reached a plateau at approximately three years after gene therapy (Figure 3).

### 3.4 Adverse Events

The patient experienced 20 AEs during the follow‐up period, of which two were serious adverse events (SAE), including a hepatic enzyme elevation at week five and a traumatic acetabulum fracture at week 16 (Table 1). The ALT elevation was categorised as a grade 3 level. The acetabulum fracture was not dislocated and required no surgical intervention. There were no bleeding symptoms associated with the fracture. Enoxaparin was introduced for thrombosis prophylaxis after the event of the fracture in week 16 and followed for three months until week 30. The majority of AEs and SAEs were documented in year 1. At week 40 an anticoagulant therapy was introduced with apixaban following a D‐dimer elevation to 3.87 mg/L (normal range 0.5 mg/L) and was continued until week 54. Glucocorticoid‐related AEs were predominantly observed in the first year, with resolution of the initial weight gain by week 55. Osteoporosis was diagnosed in the setting of the fracture and was assessed as most likely to be corticosteroid‐induced. No investigations of bone metabolism prior to gene therapy had been performed. At data cut, the patient had not recovered, but the AE was resolving. Throughout the follow‐up period (years 2–6), there were no significant increases in ALT or AST levels. No AEs related to infusion and anaphylactic reactions, as well as inhibitors to FVIII, thrombotic events or malignancy, were documented (Table 1). The absence of both traumatic and spontaneous bleeding events and the maintained normal FVIII levels indicate a successful long‐term response to valoctocogene roxaparvovec gene therapy.

**TABLE 1:** Overview of the patient's AEs experienced during the observation period.

		Year 1 (until week 52)	Year 2 (until week 104)	Year 3–6 (until week 312)
AEs		16	1^†^	1^†,‡^
SAEs		2	0	0
Treatment‐related AEs		4	0^§^	0^§^
Glucocorticoid‐related AEs		2	0^¶^	0
AEs of special interest	ALT elevation	1	0	0
	ALT elevation grade 3	1	0	0
	Infusion‐related reaction	0	0	0
	Anaphylactic/anaphylactoid reactions	0	0	0
	Thromboembolic events	0	0	0
	Anti‐FVIII neutralising antibodies	0	0	0
	Any malignancy	0	0	0

Abbreviations: AE, adverse event; ALT, alanine transaminase; FVIII, factor VIII; SAE, serious AE.

^†^ Follow‐up AE (osteoporosis) was not resolved at data cut

^‡^ Follow‐up AE (gastritis) was resolved in week 120

^§^ Follow‐up AE (D‐dimer elevation) was resolved in week 156

^¶^ Follow‐up AE (weight gain) was resolved in week 55.

### 3.5 Outcome and Efficacy of Gene Therapy

By the end of the six‐year follow‐up, the patient demonstrated sustained normal FVIII activity levels, without any bleeding episode requiring FVIII infusions (Figure 2). A decrease in FVIII activity was observed mainly during the first two years, followed by a more gradual decline or even a plateau in the subsequent years (Figure 3). Notably, these levels were maintained within the normal range.

## 4 Discussion

Valoctocogene roxaparvovec therapy provides significant benefits by eliminating the need for regular prophylaxis with either intravenous FVIII or subcutaneous non‐factor therapies, thereby enhancing quality of life. In the GENEr8‐1 study, 8.3% of patients maintained FVIII levels above 40 IU/dL (as measured by the chromogenic FVIII assay) for five years post‐infusion [21]. Our patient showed sustained normal FVIII activity levels without requiring any FVIII supplementation for six years.

Extrapolation of the most recent clinical trial data of valoctocogene roxaparvovec (4–7 years of follow‐up) suggests an estimated long‐term durability of treatment effect of 11–17 years [22]. Loss of response to gene therapy was defined as FVIII <5% and at least two treated bleeds in six months prior to return to prophylaxis [1, 23]. To date, 25 participants (18.7%) from the GENEr8‐1 trial returned back to prophylaxis [21]. Considering the subset of 17 participants who have been followed for more than four years using predictive indicators such as pharmacokinetic models, FVIII expression appears to stabilise over time [22, 24]. Noteworthy, in our patient, median FVIII levels indicate a plateau over the last three years, further supporting the favourable long‐term outcome of gene therapy in this patient.

In our patient, immune‐mediated challenges emerged, beginning with elevated ALT levels in week 5. The initial response to 60 mg oral prednisolone was insufficient, necessitating hospitalisation and treatment with intravenous methyl‐prednisolone therapy according to a standard protocol. At week 18, tapering of corticosteroids was halted, and the dose was increased to 35 mg due to a relapse of ALT elevation. Around that time, study investigators were informed that a significant number of patients experienced ALT relapse when steroid doses were reduced below 30–35 mg/day. Due to side effects of the long‐time corticosteroid therapy in our patient, budesonide was chosen as an alternative steroid therapy with fewer systemic side effects and was tapered over 25 weeks. During budesonide treatment, FVIII:C remained stable and was consistently above the reference range.

This case shows that immunosuppressive therapy may rescue FVIII gene expression in selected hemophilia A patients [15]. In the GENEr8‐1 trial, 78.4% of participants received glucocorticosteroid treatment because of increased ALT levels [22]. In 75 participants (56%), ALT elevations occurred more than once [9]. Although corticosteroids control ALT elevations and, in some patients, prevent loss of FVIII expression, their management remains complex and individualised, indicating that personalised regimens may be required to optimise outcomes. [14]. Long‐term corticosteroid use may have side effects, including osteoporosis, hyperglycemia, and increased infection susceptibility, which must be balanced against the benefits of maintaining FVIII levels. Elevated ALT levels are a common AE in various gene therapies using different AAV serotypes, as seen in clinical trials for other diseases [25, 26, 27, 28].

In our case, the individualised corticosteroid regimen with methyl‐prednisolone, prednisolone and budesonide lasted approximately 40 weeks in total. The decision to discontinue budesonide was made at week 31, following the second consecutive month of normal ALT and AST levels. Due to a stepwise tapering—reducing the dose from 9 mg/day to 6 mg/day over eight weeks, followed by 3 mg/day for an additional four weeks—the tapering process extended over 12 weeks. Given the favourable safety profile of budesonide and our intention to minimise the risk of relapse, our strategy to taper and discontinue immunosuppression can be considered cautious and conservative.

During the treatment with steroids, clinicians should monitor liver function and FVIII levels closely, adjusting steroid dosages as needed to maintain therapeutic efficacy.

The necessity and clinical benefit of prolonged immunosuppression (median time in the GENEr8‐1 trial was 33 weeks)—sometimes lasting up to a year [22]—in a significant proportion of patients with hemophilia A after gene therapy remains unclear. While the initial immune response to the AAV capsid has been well characterised as a T‐cell‐mediated reaction similar to that following viral infection [13, 29, 30], the capsid proteins are expected to be metabolised within 8–12 weeks post‐infusion [31]. Beyond that period, no target for anti‐capsid antibodies should remain, suggesting that other mechanisms contribute to the need for extended immunosuppression.

One hypothesis is that hepatocellular stress from ectopic FVIII production may play a role, as FVIII is normally synthesised by liver sinusoidal endothelial cells [32] ‐ not hepatocytes. A previous gene therapy trial for hemophilia B failed due to a Toll‐like receptor 9—mediated response to codon‐optimised DNA rich in CpG motifs, which are recognised as foreign (e.g., bacterial) DNA [33].

Notably, at the time of the bone fracture in the presence of increased D‐dimers, anticoagulant treatment with enoxaparin was given. The decision to restart anticoagulant therapy in week 40 was made on the basis of significantly increased D‐dimers and supraphysiologic FVIII activity [34, 35].

In summary, this case highlights the potentially critical role of corticosteroids in managing immune responses and thereby possibly ensuring the durability of transgene FVIII expression. The possible benefits of prolonged steroid therapy in hemophilia A patients undergoing gene therapy emphasise the need for further research, advocating for optimised immunosuppressive protocols to enhance safety and efficacy in the context of gene therapy.

## Author Contributions

K.H. T.A. and J.O. coordinated the research. K.H. and B.P. wrote the initial draft of the manuscript, analysed the results and created the figures. C.K., J.O. and G.G. recruited patients. J.O. was the principal investigator and wrote the manuscript. J.O., G.G., C.K., P.L., C.S. and U.S. made the clinical evaluation. All authors contributed to data analysis, laboratory/correlative analyses, and manuscript editing and evaluation and read and approved the final manuscript.

## Funding

This research received funding from institutional sources.

## Ethics Statement

The present study used samples from a patient participating in the GENEr8‐1 and GENEr8‐LTE trials. Both trials were conducted in accordance with the Declaration of Helsinki and the applicable International Council for Harmonisation of Technical Requirements for Pharmaceuticals for Human Use Guideline for Good Clinical Practice.

## Consent

For samples from the GENEr8‐1 and GENEr8‐LTE clinical trials, informed consent was obtained from the participant.

## Conflicts of Interest

K.H. received travel support as well as personal fees for lectures from BioMarin. B.P. reports having received grants for research from Biotest and Octapharma as well as personal fees for lectures and advisory board meetings from NovoNordisk and Octapharma. J.O. reports having received grants for studies and research from Bayer, Biotest, CSL‐Behring, Octapharma, Pfizer, SOBI, and Takeda, and travel support as well as personal fees for lectures and advisory board meetings from Bayer, Biogen Idec, Biomarin, Biotest, CSL‐Behring, Chugai, Freeline, Grifols, Novo Nordisk, Octapharma, Pfizer, Roche, Sanofi, Sparks, Swedish Orphan Biovitrum, and Takeda. G.G. has received advisory board and travel expenses from Bayer, BioMarin, Biotest, Chugai Pharmaceutical Co., Ltd, CSL Behring, Grifols, LFB, Novo Nordisk, Octapharma, Pfizer, F. Hoffmann‐La Roche Ltd, Swedisch Orphan Biovitrum, and Takeda. T.A. reports having received grants for a patient support association from Bayer, Biotest, Chugai, CSL‐Behring, Grifols, Novo Nordisk, Octapharma, Roche, Swedish Orphan Biovitrum and Takeda, as well as personal fees for advisory board meetings, consulting and/or travel support from Bayer, Biomarin, Biotest, CSL‐Behring, Grifols, Novo Nordisk, Octapharma, Pfizer, Swedish Orphan Biovitrum and Takeda. P.L., C.S., and U.S. report no conflict of interest.

## Data Availability

The data that support the findings of this study are available on request from the corresponding author. The data are not publicly available due to privacy or ethical restrictions.
